# Self-Perceptions and Behavior of Older People Living Alone

**DOI:** 10.3390/ijerph17238739

**Published:** 2020-11-24

**Authors:** Jesús Molina-Mula, Julia Gallo-Estrada, Antonio González-Trujillo

**Affiliations:** 1 Nursing and Physiotherapy Department, Universitat de les Illes Balears, 07122 Palma (Illes Balears), Spain; j.gallo@uib.es; 2 Center for Innovation and Development in Nursing and Physiotherapy of the Balearic Islands of the Nursing Union (SATSE-CIDEFIB), 07003 Palma (Illes Balears), Spain; antonio.gonzalez@uib.es

**Keywords:** elderly people, self-perceptions, Bourdieu, ethical issues, public policy

## Abstract

It is currently acknowledged that older people prefer to live in their own home, even if they are lonely or disabled in some way. The factors that condition aging among older people members of the population living alone include the following: the existence or absence of a social network, gender, the home or place where they live, their capacity to function, and welfare and health resources. The main goal of this study was to explore the perceptions of older peoples over 75 years old about adaptation strategies and the social, gender, physical autonomy, and socio-health resource factors that determine their permanence at home. The authors used a qualitative methodology, within a critical social framework, based on the theories of Pierre Bourdieu. When the interviewees’ discourse was analyzed, four main categories were evident: (a) “A desire to stay at home”, (b) “Changes and every-day aspects of domestic life”, (c) “Reliance on social and family assistance”, and (d) “The use of social services and resources”. In synthesis, the participants questioned the benefits of the type of home life offered by members of the family. They believed that, in some cases, this option did not overcome the problem of loneliness or the need to hire assistance. The findings of the study revealed that one needs to dispel the notion of geriatric care as a form of charity, and to distinguish between the activities of caring, providing support, and offering companionship to someone. It is important to identify products designed for older people who might live for a long time.

## 1. Background

The study of old age is a recent phenomenon. Aging is a social issue that encompasses how we organize ourselves and how we respond to new challenges [1]. Indeed, aging today constitutes an achievement, thanks to our longer life expectancy, but it also represents a threat in terms of tackling the consequences. Some demographers refer to this process as the democratization of survival in allusion to the fact that almost all newborn babies will have the chance to live through all stages of life [2]. This democratization process has been widely developed mainly in Spanish literature [3,4].

Professionals and public opinion indicate a preference for community support as a way of allowing older people to stay in their own homes as long as possible [5]. The responsibility for problems associated with dependence should ideally be transferred from the family to the community, and assistance from the medical domain to the social realm [6]. In other works, the family should not only be held responsible for the dependency problems of older people, but the community with certain support or resources should assume this responsibility. Similarly, the healthcare model should not only be based on a biomedical model focused on the disease, but should also be based on a coordinated and person-centered social model.

Information on living preferences points to an increase in the number of older people who live alone. The proportion of older people living alone in Europe ranges from 50% in Denmark to less than 20% in Spain, Greece, or Portugal [7]. It should be emphasized that living alone does not necessarily imply being independent [8]. According to the National Institute of Statistics of Spain, 608,000 older people over 65 years old who live alone have disability or problems in activities of daily living [9]. For this reason, it is necessary to understand their needs to improve their quality of life.

The risk of poverty among older people living alone in Europe is higher than that among those living with another person. In countries with a higher level of social and economic development, fewer older people live with another person [10].

What is really a determining factor is not so much living alone as is being an older woman living alone. Women in developed countries are approximately two and a half times more likely to live alone due to their greater life expectancy and because of their habit of finding a partner who is older than them [11]. In countries where their social role is largely dependent on their status as a daughter, wife, or mother, living without a spouse plunges woman into a state of vulnerability, if not marginalization [11].

The factors that condition aging among older people living alone include (a) existence or absence of a social network, (b) gender, (c) the home or place where they live, (d) their capacity to function, and (e) welfare and health resources.

Despite the different ways of looking at old age, there is consensus among the authors that from 75 years of age, older people have more needs and more risk factors. Neugarten [12] established two categories: before and after 75 years.

The data show that the risk of poverty among older people who live alone in Spain, as in the rest of Europe, is higher than among those who live in two-person households. In Spain, almost half of these older people who live alone have incomes below the poverty line. However, living alone is not as decisive as being an older woman living alone [13].

Although aging involves changes inherent to that stage, these changes act differently influenced by different factors. The risk of suffering from disability increases as educational level decreases. Studies are considered an indirect indicator of economic and social position; in addition, instruction is related to capacities and use of resources [8].

Data from the study carried out in three European countries by Zunzunegui, Rodriguez-Laso, Otero, Pluijm, Nikula, Blumstein, Jylha and Minicuci [14] corroborate that there is a generalized perception that participation in social activities by older people is associated with a lower risk of dependence in the future and with greater functional capacity for the development of BADL (basic activities of daily living). The results of other studies indicate that the social network shows a protective effect in the transition from functional limitations to disability [15,16].

Gender marks a difference: Men feel the impact of the loss of a spouse more than women. Thierry [17] calls it “Widowhood shock or broken heart theory” (“Choc du veuvage ou théorie du coeur brisé”, name of the original source in French). Women find partners in fewer cases as well as avoid the need to live with their children/relatives [18].

One can attribute an older person’s integration into another home to members of the family regrouping or a situation of dependence [19]. For older people, their own home is the place where they cope best, and they are reluctant to leave [13]. Less sense of isolation is associated with safe homes close to relatives, and to the existence of services and transport [20].

Regarding an individual’s capacity to function, aging is an admittedly two-directional interactive process between the body and background environment [21,22]. A disability is an obstacle in a person’s capacity to function, and their ability to meet the demands of their immediate and local environment [23].

With respect to this two-directional interactive process, referring to the body, for older people, the home is presented as the place where they can best be managed, since it has the order that they have created; for this reason, they are reluctant to abandon it. Unknown spaces attack, due to the amount of new information they may contain, or because of the new skills they demand [20].

The home environment has the capacity to articulate the relationship exchange of households with the outside. Carp [24] collected definitions that could be found in older people that have in common the fact of recognizing a progressive deterioration that makes them more affected by their environment than younger people. Consequently, various environments should support them to compensate for these losses. Older people value satisfactorily, according to Casas et al. [25], adequate and safe environments and highlight the importance that the environmental context is for them passable, clean, well lit, well connected, and has usable urban furniture.

The configuration of the city is a factor to explain loneliness. Medina and Cembranos [26] considered that for older people, the city is not accessible; it is not to master it, understand it, or establish relationships. In the city, proximity is lost; it is difficult for their neighbor to be interested in their mother, or to talk on the street. Faced with this loss of space for personal relationships, older people isolate themselves at home.

Considering the characteristics of older people can establish the difference between autonomy and dependence. The environment of the home must offer conditions so that the basic and instrumental activities of daily life can continue to be carried out normally, although it is necessary to have certain support. Various studies have analyzed the relationship between housing and the risk of isolation [21,27,28]. Everyone agrees that safe homes, close to the family, with adequate services and transportation facilitate social interaction. The World Health Organization (WHO) [1] proposes policies that it has called, friendly, with older people, enabling those with disabilities to stay longer in their homes and participate fully in the life of their community.

Essentially, it is hard for older people to find out about services designed to boost their independence and understand how they work. In some cases, they find it hard to acknowledge that they need support [19]. The reviewed literature shows that older people prefer to live alone. This work aimed to identify the resources and care required to respect these preferences.

The main goal of this study was to explore the perceptions of older peoples over 75 years old about adaptation strategies and the social, gender, physical autonomy, and socio-health resources factors that determine their permanence at home.

## 2. Materials and Methods

### 2.1. Theoretical Approach to the Concepts of the Theory of Bourdieu

It is possible to interpret “being older and living alone” as a socially constructed human activity, in which social structures determine its development and the value it is attributed, but which are in turn interpreted and reorganized by the agent. Residential solitude of older people is linked to social constructs such as gender, age, and the very concept of solitude and, besides, this construction depends on the particularities of each context such as socio-educational values. This perspective allows a glimpse of people’s social and family system and the smallest modification may bring about great transformations. In this sense we will use, at times, the Bourdieun reference of “sociology of action” [29].

The theory of Pierre Bourdieu stands out as an attempt at overcoming the traditional duality present in sociology between social structures and objectivism (physicalism) as opposed to social action and subjectivism (including interpretation or hermeneutics) [30]. For this, he used two new concepts, habitus and field, while reinventing an already established one, capital; which he diversifies and equips with a new, transforming dynamic potential. To a large extent, in fact, what it is about is seeing how the different types of capital—which define the possibilities of the different social positions in the corresponding fields—are transformed one into the other [31]. Something which, as we shall see, led us to see how the social construction of gender (and the different competencies it entails) and the accumulated cultural-educational capital, together with the corresponding symbolic capital of recognition and self-recognition, reveal the attitudes and different likelihoods of obtaining high degrees of autonomy (real or perceived, which do not always necessarily coincide) in older people living alone; all of this, obviously, embedded in a certain prior domain of the social capital obtained, accumulated, and handled [32].

Hence, the nucleus of this research are the needs that older people living alone have to cope with. Taking the home as the field will help us to study the relationships and intervention modalities that are found in our unit of analysis. The different types of capital will help us to understand the meaning of what the agents do, according to the place they occupy in the field. Furthermore, the analysis of the habitus of the agents that perform the activity within a certain structure such as the home shows us different ways of being in the world and of understanding the reality they face daily [33]. These aspects reveal divergences or conflicts, thus creating what Bourdieu calls a certain type of symbolic violence [34].

The formula {(habitus) (capital)} + field = practice, which Bourdieu proposed, was the starting point in this theoretical development. The concepts of Bourdieu’s thinking help us to find out about some of the mechanisms governing the residential habits of older people and the dynamics that are constructed around this situation. It also enables us to identify what dynamics bring about tensions between older people and their families [32].

Bourdieu recognizes that individual and group actions, in an area such as the home, cannot be explained as individual behaviors without considering the underlying structure, but rather as actions with a cultural influence of traditions and objective structures within society [35].

Bourdieu seeks the social order hidden behind the symbolic order in order to rationally explain social structures and spaces; he aims, thereby, to construct a more realistic view of the space studied. The agents, according to Bourdieu, reproduce practices in accordance with the position they occupy in a social space. Man is the product and producer of social reality through rules that make up the habitus [36].

Habitus is a concept that plays a very important role in the construction of the social world. Bourdieu [29,33] understands habitus as the set of dispositions whereby subjects perceive the world and act in it. It is “the internalization of externality” and the “externalization of internality”; it is society in the body and in the mind, which makes perception, appreciation, and social action possible for subjects, instilled by a particular social context. The habitus for Bourdieu is a “structured structure” capable of operating as a “structuring structure”; a set of incorporated, durable, transposable, and transformable dispositions that allow the subject behaviors and attitudes while limiting their scope of action [37]. This fact becomes evident when we are immersed in a medium, different from ours, whose rules of play we do not know [30].

The habitus is the response of individual experience and collective history; it enables us to explain some characteristics of social life, not only as a result of the actions of individuals, but also based on the influence of history, tradition, and people’s principles. Unconsciously, individuals incorporate behaviors into their lives by imitation [38]. In order to analyze this influence, Bourdieu and Passeron [39] used the term inculcation to refer to the pedagogical action carried out in the family space. It is precisely this interest in social reproduction where social differences and structures are reproduced that provides us with an explanatory instrument for our research, most particularly the components of the habitus that refer to training, education, and culture, often independently of level of officially achieved formal education [40].

The way in which the practices of subjects are organized according to their perceptions is based on habitus. The choices of social agents obey the practices internalized by the social group in which they have been brought up. These ways of behaving will in turn be adopted by new agents. The habitus provides the subject with the necessary skills and values to integrate in a group; it provides the ability to act without the need for establishing a plan of action. Actions are the result of dispositions incorporated in the course of a trajectory [41].

The habitus is considered, on the other hand, an element that hinders change in behaviors and, as such, is useful in analyzing residential solitude in older people and the relationship of older people living alone with their social and family network. As Granés [42] propounds, individuals will operate according to external factors and not according to their interests or values; and their choices will be the result of their background and their social conditions. In this same sense, Angus et al. [43] highlighted the impact entailed by incorporating the logic of the health sphere in the recipients of home care into the arena of the home. The intervention of different practitioners in the home over a long period of time brings about changes that transcend the limits of the home in which families also intervene.

Habitus and field are intimately related, thereby, the field structures the habitus, which in turn contributes to building the field as a world endowed with meaning [36]. Although the actions of individuals may be governed by the field rules, not in all fields or for all participants is this character conscious, homogeneous, and unique; the orientation given by the habitus imposes a strategy. Agents are not wholly the author of their practices; the social force acts invisibly on them. Thus, this explains the fact that sharing a social position produces similar behaviors [29].

Bourdieu specifies that fields are neither autonomous nor comprehensive; in fact, society and social action move from one to the other, they form subfields and the different capitals circulate among them. Hence, the residential solitude of older people will be influenced by family structures, environmental, and personal factors. However, society’s structures determine how residential solitude and old age are conceptualized.

One’s position in the field studied depends on the capital recognized by society itself; it is the sphere in which there are encounters between individuals that enable opinions to be confronted with power struggles and relationships that are often asymmetrical. For Bourdieu [31], in these struggles, the limits of the field also come into play as who may go in, who has the power to decide, what the power shares are, and how they are shared out.

Thus, the home of an older person may be considered a field of study that functions more or less autonomously with its own laws; a complex sphere, made up of the older person, the family who without sharing the home provide care, the neighbors and friends and formal carers who carry out their work in the home (therefore, the field of “home” acquires its full social dimension—its context—not being restricted to the immediate physical environment). In order to venture into this universe, the internal codes and rules must be known [31].

Capital can take on different shapes that are determined by the field in which it will be used; it can be exchanged or used to improve one’s position in the field. Bourdieu did not consider capital as an economic mechanism. However, he recognized the importance of economic capital and the resulting class structure in a modern society. The name capital is given to the resources brought into play in the different fields: economic capital, cultural capital, and social capital. For this reason, the field also works as a market of all goods material or symbolic [29].

Bourdieu used the concept of “social capital” to refer to the advantages and opportunities that people obtain by being members of certain “communities”.

Bourdieu [33] considers cultural capital to have three typical states, distinguishing for each state a modality of acquisition and of transmission. Cultural capital can exist in an embodied state, that is, under the form of the organism’s durable dispositions; it cannot be delegated, must be acquired; and dies with the biological capacities of its bearer. Its method of acquisition makes it appear as innate property. Cultural capital in an objectified state is found in the shape of cultural assets such as books, instruments, sculptures, paintings, machines, etc. Finally, capital in an institutionalized state confers the bearer a conventional, constant, legally guaranteed value.

Bourdieu [33] introduces symbolic capital as a fourth type of capital. These are certain properties that seem to be inherent to the person such as authority, prestige, reputation, credit, fame, notoriety, honor, good taste, etc., properties that can only exist insofar as they are recognized by others. Understood in this way, symbolic capital “is no more than the economic or cultural capital that is known and recognized”. Respectability of an older person at the top of the patriarchal family seeks in present day society its space in the new family models. Symbolic capital exists insofar as its authority is recognized by others as a value. From the moment in which in our research the concept of autonomy to live alone includes physical dimensions related to the objective situation of health, and also self-perception and external perception (socio-medical and family networks), to a large extent, the symbolic capital becomes involved in shaping the opinions others have of older people and of them with respect to themselves: likewise we see here the relevance of working at looking into how some capitals (economic, social, cultural) come to be transformed into others (among each other and into symbolic capital) and how this results in a greater or lesser capacity of acceptance/being accepted as an autonomous person.

Knowing why older people are the way they are, providing a more integrating view, prioritizes the analysis of the phenomenon as opposed to its description and enables us to explore and question some aspects of the identity of older people that are hardly discussed nowadays.

### 2.2. Methodology

A qualitative method was used for the study based on the theories of Pierre Bourdieu, who introduced potential fields of research on the reciprocal influences of status, social structure, and role system in the make-up of older people living alone as a result of multiple processes of social interaction. Above all, he broke new ground in terms of the concepts that he coined for a field (in our case, the home and social context), social capital (social networks), cultural capital (knowledge and a person’s status as a result of their upbringing), and the *habitus* (set of generative schemes from which subjects perceive the world and act in it. These generative schemes are socially structured: they have been shaped throughout the history of each subject and involve the internalization of the social structure, of the concrete field of social relations in which the social agent has been shaped as such).

The study was conducted in two stages: (a) a pilot stage, where two interviews were held to draw up a basic question matrix directed at older people above the age of 75 who lived alone to ensure that specific proposed objectives were met; and (b) an exploratory stage in which 17 interviews were held with older people aged above 75.

The participants comprised women and men over 75, living alone in Mallorca. We used purposive sampling methods excluding people who were not healthy enough to communicate in Spanish or Catalan, or understand these spoken languages. Among the selection criteria considered were: (a) Educational level (higher level with university studies, intermediate level with secondary education studies, and basic level with primary education studies); (b) social network (with family network, with social benefits and without support social networks), and (c) autonomy in activities of daily living: according to the Lawton & Brody on instrumental activities of daily living scale (I.A.D.L.) (more than eight independent person and less than eight dependent). These criteria allowed for quota sampling to ensure the inclusion of participants in each category.

A total of eight men and eight women above the age of 75 were recruited. Subsequently, we took a decision to interview one more person, given the complexity of interviewing a man aged 103. We recruited participants using the “snowball” technique, requesting their voluntary participation.

Discourse analysis was used as a research method, based on the social critical references and the concepts of Bourdieu’s theory. Any of the phases of the research process can be conceptualized as analytical, since from the formulation of the problem, the researcher is dissecting the phenomenon, and the task of posing and solving questions does not stop until the presentation of results.

This proposal, which could be defined as “healthy and prudent eclecticism” [44], must be considered since, on occasions, paradigmatic positions held as opposite (even by their main developers) can be complementary, as each one of them addresses different aspects of the problem studied.

The analysis is enriched with information arising from the relationship with the informants or from events produced during the field work. In this work, the analysis responded to two sequentially interrelated parts by means of a deductive coding process based on already established theories about residential loneliness strategies, and an inductive analysis to ensure that the data were sufficiently broad to produce new contextualized knowledge.

For the initial identification of thematic areas, the texts of a series of interviews were compared trying to give a common denominator to a set of interview fragments that share an idea (for example, current housing). Under that code, we met a varied verbatim “I do office work”, “I don’t like cooking”, and “it’s like a student flat” phrases and fragments that contained evaluations of the older people about the type of home and their relationship with it. The category groups, according to the researcher, were a set of contents referring to the same phenomenon.

This type of encoding has been called “open encoding” [45]. The goal of this encoding is to open the query. The analyst remains open to concepts of a higher degree of abstraction suggested by the data itself, with the aim of identifying the most relevant categories, overcoming the possible confinement of established analysis categories.

The result of the analysis of the texts is, as Conde [46] points out, the discourse, a theoretical construction that involves analyzing its components, how they are structured, the relationship these have with the social context, with the research subjects, and the type of social reality that they help to build.

Discourse exists in relation to another discourse that it tries to approximate or from which it tries to differentiate itself. In reference to the production of the discourses, the authors highlight their social character that goes beyond the subject itself and quotes Bajtin paraphrasing: “The life of the word consists of passing from word of mouth, from one context to another, from a group social to another (…) in this way the word does not forget the path traveled and cannot completely free itself from the contexts of which it has previously been a part” [46].

This study was authorized by the Research Ethics Committee of the Balearic Islands with the code IB1974PI-11 on 12 July 2017.

## 3. Results

**Figure 1:**
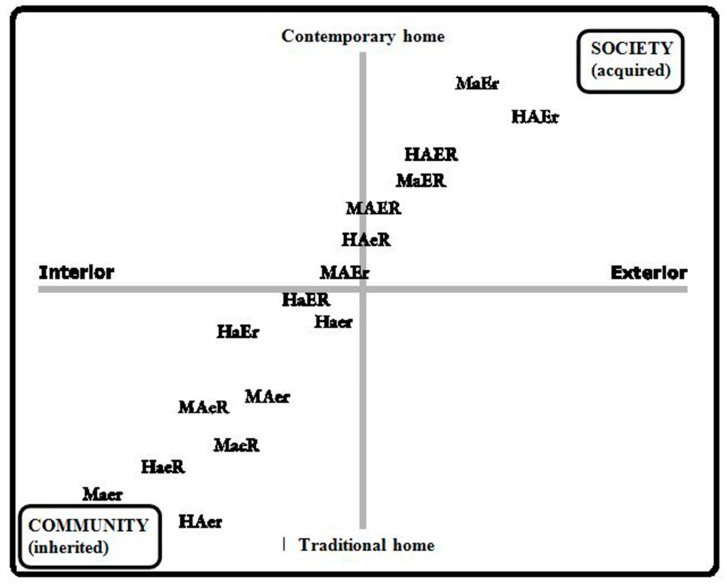
Map positions of respondents. MAER: woman, with autonomy, higher education, with social and family network. MAEr: woman, with autonomy, higher education, with poor social and family network. MaeR: woman, with autonomy, basic studies, with social and family network. MaER: woman, without autonomy, higher education, with social and family network. MaEr: woman, without autonomy, higher education, with poor social and family network. MaeR: woman, without autonomy, basic studies with social and family network. Maer: woman, with autonomy, basic studies, with limited social and family network. HAER: man, with autonomy, higher education, with social and family network. HAEr: man, with autonomy, higher education, with poor social and family network. HaeR: man, with autonomy, basic studies, with social and family network. HaER: man, without autonomy, higher education, with social and family network. HaEr: man, without autonomy, higher education, with poor social and family network. HaeR: man, without autonomy, basic studies with social and family network. Haer: man, with autonomy, basic studies, with limited social and family network.

Although the portrayed figure is not scaled (Figure 1), the study’s participants were positioned between the ends closest to community values—as traditional, rural, inherited phenomenon, generated spontaneously (inherited *habitus*)—and social values, as artificial, rational, urban, learnt grouping phenomenon (acquired *habitus*). The lives of this group of older people are notably affected by social influences derived from their experiences during their youth and adulthood. The men were self-taught in their work, and the women gave priority to their roles as wives and mothers as opposed to professionals, with all the women being just housewives for relatively long periods of their lives.

Figure 1 represents what Bourdieu suggests as the schemes of thought, perception, and action, which are revealed based on a certain social genesis that determines the acquisition of certain habits that remain anchored to the spaces of the social field or groups in which the agent operates.

In our study from a first reading in order to reflect on the possible modifications to be made, we prepared an interview map in relation to the home/dwelling dimension—the current or traditional aspects of the home—that could be related to adaptation strategies and stay at home, in addition to the open nature or not of the same that could determine their experiences with the social network and resources.

Although the graphic representation is not metric, the distribution of the subjects of our study supports the idea that social patterns are not random. According to what we assumed at the beginning of the work, the interviewees were positioned between the extremes closest to the values of the community, as a structure created spontaneously, traditional, rural, and inherited; or those closest to social values, what are acquired as an artificial, rational, urban, and learned grouping. When observing the opinion system of the interviewees, the influence on residential attitudes of the home itself as an identity space in which to reside is verified.

Four overarching categories emerged when we analyzed the interviewees’ responses (see Table 1, where they are presented *verbatim*).

In the “stay in one’s own home” category, the following codes were established: (a) *The home as a place of freedom and power*: The importance of one’s own home lay in the fact that it is the place where one has lived, and where one exercises the most control, freedom, and capacity for self-realization. (b) *The home as a form of identity*: Two attitudes were observed; a traditional attitude that viewed the home as an inherited place, with unchanging, affective connotations only perceived by the family; and another a more up-to-date one, where it was seen as a comfortable, open place for enjoying memories, filled with indications of a modern, technological life, in allusion to social constructions. This group preferred to hire help for domestic work. (c) *Concern about the future:* In terms of their lifestyle, they preferred to concentrate on today rather than thinking about a non-existent future, preferring not to anticipate losses, and avoiding planning for the future. (d) *Living alone as a way of life:* Loneliness, on the one hand, was seen as an imposed situation. It was accepted (with an effort), however, the person worried about the availability of support from social and family networks. On the other hand, it was a way of life that had positive aspects in terms of freedom, to the extent that they preferred not to live with someone else. (e) *Different attitudes according to gender*: Gender marked a difference when it came to living alone. Men living alone had difficulties in carrying out activities in the home, while women had difficulties handling things outside the home. (f) *Relation between personal perceptions of independence and over-estimating abilities:* Being independent was perceived by more traditional older people as not needing help. They tended to over-estimate their abilities, hiding their losses from others. If they could get by, they did not ask for or hire help. For others, their abilities were associated with their capacity to successfully adapt to the changes that accompanied aging. If they had support, they used it.

In the “changes and every-day aspects of domestic life,” the following codes emerged: (a) *Strategies to stay in the home*: The older people felt greater security and more sense of control in familiar places. (b) *Self-care as a way of continuing to live on their own:* More traditional older people preferred to cook as a means of conserving their way of life. More modern older people preferred not to cook.

The codes in the “Reliance on social and family assistance” category were: (a) *Traditional family—care as a moral duty*. Traditional families considered themselves to be the main support of the older people. In the absence of family members, certain neighbors ranked next in importance. Care was considered a moral obligation, and the possibility of hiring help with domestic tasks was not contemplated. (b) *Alternatives to traditional care: Current conception of care for the older people* consider friends, neighbors, unmarried partners, and others to form part of their network. These older people did not consider caring as a family obligation.

In “The use of social services and resources” category, the following codes emerged: (a) *Their attitude to health care professionals*: The older people regarded assistance by health care professionals as occasional, and primarily saw them as the suppliers of medicine. (b) *Technology and backup tools for people:* Older people were aware of support systems but felt unable to carry out the complex formalities needed to request them. The best-known, most widely used tool was call alert telecare. (c) *Alternatives to living in their own home:* When they cannot continue to live alone at home, they considered two alternatives: living in an old-age home, overcoming any negative connotations that it may have, or staying with a relative, generally their children.

## 4. Discussion

Conversations with older people fluctuated between demonstrations of their ability to live in their own home as they wished, similar to the study of Abellán García et al. [7]. In our study, we observed differences between what an older person said and thought they would prefer to do when they were no longer independent, and what relatives said and thought when alternatives were offered to living alone. A closer relationship between the carer’s expectations and those of the older person regarding the latter’s ability to cope with everyday activities on their own is associated with higher levels of wellbeing [47].

Some studies have demonstrated a link between carrying out everyday activities, self-esteem, and perceptions of one’s efficiency [48,49]. Although in most cases the interviewees did not choose to live alone, two different attitudes could be perceived. It was seen either as a situation they accepted effortfully or as a situation chosen for them, as in the study by Bermejo Higuera [49].

In our study, the interviewees considered their abilities as inherent, and tended to show a conformist attitude. In the case of those who believed that abilities were largely acquired, the concept of adaptation to change emerged in conversation with them. Bourdieu used the term strategy to refer to practices that facilitate acknowledgement of the difficulties imposed by the field, and the *habitus* to make this change [50].

Older people tend to minimize problems, lowering their everyday demands and procrastinating in finding solutions, in keeping with studies by Ruiz-Jiménez et al. [51]. Older men and women agreed that living alone was easier for women than for men. Quantitative studies show that men are more dependent when it comes to carrying out daily domestic tasks, although this is a cultural dependence [52]. Failing to do domestic tasks is not related to their abilities, but to the clearly observed distribution of gender-based and generational roles, particularly in their adaptation to everyday life when they lose their spouse [53].

It is the traditional family head, based on patriarchal models, who defines the logic of the field, and they foster a symbolic system of family relations to justify the authority they hold [37]. In a traditional social network, older people consider that caring for its members is a moral duty. This finding coincides with the study by Adelantado et al. [54], who stated that public services play a minority role in care, and that relatives become responsible for looking after an older family member when the latter becomes more dependent.

In contrast with the traditional notion of a family where children are obliged to care for their elders, new prospects of planning for old age have emerged, where the family is not the only supplier of care. This is an emerging concept that tends to move away from the results of studies like the European OASIS [55], where Spain stood out in its clear preference for family care, unlike countries with more developed welfare systems like Norway.

Older people have little contact with health care professionals; and when they do, it is mainly for pharmacological treatment. One problem that was highlighted in our study was the distance from their home to the health center, also observed in the study by Compán-Vázquez and Sánchez-González [56]. Older people regarded health care professionals as providing fragmented assistance, and they could not identify the agent providing the care. This finding is shared by several authors [40,55,56].

The “complicated to request” and “slow at providing assistance” code explains the scanty interest shown by older people in resources aimed at facilitating everyday domestic tasks. In this respect, López Doblas and Díaz Conde [57] pointed out that criteria for granting such assistance must be made more flexible, its coverage extended, and forms of assistance increased.

Studies by Martínez-Villarreal et al. [58], and López Doblas and Diaz Conde [57] also reflect the additional difficulties for this specific generation of women due to poverty, their low level of education, social isolation on many occasions, and fewer calls for assistance.

The repercussion that family transformations have on the care of older people are known and are thus found in the literature [27,57,59,60].

However, although the idea that older people prefer to live alone is widely accepted, on exploring the field structures in our study, it is evident that the actions and relationships derived from residential solitude significantly modify the actions arising from the habitus of this generation of older people [37].

Older people see their home as a social field with its own logic and particular relationships, interests, resources, and practices. Although this acceptance of habitus can often imply the acquisition of a new attitude and not only the defensive reproduction of preceding ones (already incarnate) [33].

The discourses of older people revealed the perception of the home as an identitarian space. This aspect bears a relation to the tendency to perceive it as having so many comforts that they have no intention of repairing them [61]. For one sector of older people, the home continues to be a territory governed by traditional rules; for others, it is a modern, open space where the public and private come together.

Discrepancies could be seen between what older people said and thought they prefer to do when they were no longer autonomous, and what relatives said and thought when they offered alternatives for the older person not to live alone. In this sense, Bourdieu [29] considered it of the utmost importance to identify the location and trajectory of the agents in order to understand the logic of their practices. The family is aware of the repercussion that their stance has on their practices, but is unaware that what they do or think is a cultural construct.

Although for most older people, the fact of living alone was not chosen, in their discourses, we saw two different views concerning solitude: as a situation embraced with effort and as a chosen reality that was valued as positive.

Bourdieu [30] highlighted the relationship of an individual’s profession with social class as a tool of influence and power; however, retirement levels out these professions, with the capital linked to professional contents (cultural-educational capital) losing efficiency; this “professional capital”, in fact, comes to lose efficiency in that it is what is established as a content of the specific work subfields which, after retirement, cease to be effective. Another question is the part of the symbolic capital (recognition) that certain professions and jobs confer and which, undoubtedly, may remain after the disappearance of their practice (prestigious qualifications; socially well-considered professions...). To a certain extent, they are incorporated into the habitus [31]. Older people worry about the need for care; they perceive dependence as a loss of freedom.

Making the older person’s home their field is conditioned, on one hand, by the knowledge of the family agents that is learnt from without, and on the other hand, by the actions of older people, depending on their habitus, which is derived from their experience.

In our study, older people who considered their abilities were inherent to people, tended to show an attitude of conformism toward the situations they happened to live. Among those who considered abilities were to a large extent acquired, there emerged a discourse of adaptation to change. Bourdieu used the term strategy in relation to the practices that make it possible to recognize the difficulties imposed by the field and the habitus when it comes to making a change [50].

Under this acquired nuance, older people and their families consider functional capacity as not related to physical traits, but rather to an attitude toward life; this helps explain the tendency of the generation of older people to minimize problems and put off solutions.

Every change implies modifying the structures of the field and the habitus of the agents [62]; this process will depend on the necessary disposition of the cultural, social, and symbolic capital to cope successfully in new fields.

Relatives share the idea that new technologies will make it easier to stay in the home, while the manufacturers of all sorts of products consider older people potential customers.

Not knowing to what point an older person is able to live alone is a worry shared by relatives and the older person to the point of recognizing that it is an issue they avoid thinking about due to the ambivalence of loss of freedom and power at having to live with their family, or loss of identity at going to live in a residential home; which is a discourse that has materialized due to the origin of today’s residential homes: institutions which, by seeing old age from the point of view of vulnerability and under the argument of protection and care, have built up their conception with elements of discrimination.

The feeling of duty toward caring is solved by taking them into their home. The data from our research indicate that older people consider that in some cases this residential option does not solve either loneliness or the need to hire help, as the time dedicated to work for some relatives is indisputable.

Older men and women agree that living alone is easier for women in relation to the activities of daily living than for men. Likewise, from our analysis, it follows that, among older people, the fact of not participating in household chores is not related to ability; the implication in the home has always been and still is related to the pronounced division of roles according to gender and generation, which is made especially clear in their adaptation to daily living after losing their partner [53].

Gender, along with age and family ties make up a system of social hierarchy with an asymmetrical power relationship. Since the principles upon which an individual constructs his/her social identity and the representation of the functions that correspond to each gender are positioned in relation to the parents [34], it is no wonder that power relations are established between the position in the field of the new family—of reproduction—and the one occupied in the family s/he belonged to; more specifically between the accumulation of capital of the carer and the person cared for.

With respect to social-family help, a traditional understanding can be seen by several older people, for whom their representation of a possible carer is mainly associated with the women in the family. This traditional idea is shared by the sector of women with middle and basic schooling, housewives, and service industry workers between 33 and 68 years of age. Whether or not an older person continues to live alone revolves in part around their social network, on which they lean in situations of difficulty.

The head of the traditional family as an inheritance of the patriarchal model, even nowadays and especially in the generation of the older people in our study, is the person who defines the logic of the field and has been the promoter of a symbolic system regarding family relationships that justifies the authority he holds [31,38]. However, the power held by the older person requires the complicity of the family for it to be exercised and is transformed when their location in the field is changed (needs to be cared for).

In a traditional understanding of social networks, older people and their families consider caring for their members as a moral duty in which women (even in-laws) are the main players. More conservative older people try to continue being authoritarian, making the family feel bad if they are not cared for. Bourdieu [34,35,36,37] emphasizes the properties that individuals incorporate in the fields, which hide gender demands, social class or generation; in our study, the struggle of men and the older people generation to retain their power as opposed to women and the relatives’ generation was evident.

In a current understanding of social network, there emerges new perspectives of planning old age—shared by older people produces changes in the demands, among whom in our study there appeared an emerging discourse with a tendency to move away from the data in the European study OASIS [55] in which a clear preference to being cared for by the family when needed was highlighted in Spain; unlike countries with more highly developed welfare states such as Norway. This change is also likely to reflect in part improvements in the welfare state in our country.

The weak relationship between older people and the healthcare team and the fact that the motives of the relationship are mainly derived from pharmacological treatment stands out; there is also a perception of fragmented care by the healthcare team and the provider of care was not identified. This finding is shared by several authors [63,64].

Our study shares with others such as Martínez-Villarreal, Rodríguez-Ruiz, Ramírez-Llarás, García-Uso, Fabregat-Casamitjana, Fusté-Vendrell [58], and López Doblas [57], the added difficulty involved for this generation of women living alone to obtain socio-medical support; poverty, a low level of education, and social isolation which are frequent among them explain the scarcity of requests for care.

In short, as far as continuing to live alone in the home is concerned, according to our study, this should be analyzed on the one hand by considering the influence of the habitus and the cultural and symbolic capital of the subjects in adopting this state; and, on the other hand, the weight that family and social structures have in this residential solitude. At the same time, it is essential to consider the internal rules that define the limits of the home.

## 5. Conclusions and Implications

The majority of the older participants questioned the benefits of living with family members. Many of them were doubtful that living alone was better due to its positive effect in their autonomy and ability to make decisions. The majority of the older people believed that moving to live elsewhere could be associated with undesirable situations like dependence, abandonment, or being uprooted. Many of them thought that, in some cases, moving to live with family did not solve the problem of loneliness or the need to hire help, since some families have to work.

Research is needed aimed at understanding the relationship that health care professionals forge with older people to do away with the idea of care being charity, while also differentiating among the activities of caring, offering support, and providing companionship for someone, both at home and in institutions.

## Figures and Tables

**Table 1:** Categories and codes from interviews with over 70 year olds living alone.

Category “A Desire to Stay at Home”		
CODE	DEFINITION	VERBATIM
**The home as a place of freedom and power**	The home is important because it is the place where they have lived, where they conserve the most freedom to do what they want. At home, they do not have to give explanations or be forced to take orders.	*E 13: I like being alone a lot and doing whatever I want.* *E 16: When you close the door (…), everything is shut out. You’re inside and you’re the boss, the one who rules the roost there.*
**The home as a form of identity**	The presence of two attitudes to the home: one traditional view, where it is seen as a person’s own inherited home to be passed on to subsequent generations, with an affective significance for the family alone. Tasks are not delegated when it comes to caring for the home. With the other more up-to-date approach to the home, older people see it as a comfortable physical space with social connotations, where they can enjoy their memories. It is an open place for coming and going, with lots of modern touches. When it comes to domestic tasks, hired help is preferred.	*E 16: The day I get a place in an old people’s home, I’ll use it, but purely as a residential base (…). Yes, to live and, logically for food too, but the rest…* *E 7: The home is very important. It is a great refuge, filled with memories, brimming with things evocative of the past.*
**Concern about the future in terms of the type of homelife**	They tend to live for today, particularly those who are older, rather than contemplating their future non-existence. They are aware that, to a greater or lesser extent, tomorrow will bring disabilities and they tend to live in the present. Their perception of the future changes the value that is placed on time.	*E 7: Living alone will become increasingly complicated (…). I don’t even want to contemplate it. Needless to say, one day, if I get worse, I’ll have to think about doing what many older people do, finding somewhere.* *E 14: Everyone my age is dead (…) I saw one in a wheelchair the other day (…). You have to live for today. You don’t think about tomorrow because it’ll make life a misery.*
**Living alone as a way of life**	They avoid thinking about the possible drawbacks of living alone (even though it is a worry for all of them). Living alone is seen as an imposed situation which happened unexpectedly and which they accept with an effort. In such cases, there is a greater concern about the availability of social and family support networks. Being alone is also perceived as a way of life, with positive connotations in terms of freedom, to the extent that they prefer not to live with a companion or even with the family. Possible problems that might emerge when living alone worry them, rather than actually being on their own.	*E 14: When I ended up on my own, I wanted to kill myself, I wanted to kill myself, kill myself (…) I aged, I didn’t go out (…) and, when it’s 6 o’clock in the morning and the sun rises, this bird is already singing and calling me (…) animals keep you company.* *E 11: I’d feel very uncomfortable if I was accompanied all day (…) a person’s presence, now my daughter’s coming and I’m very pleased (…) but, understand me, it’d complicate my life a lot if she came to live here.*
**Differences according to gender**	The recognition by the generation in question that gender makes a big difference in the activities carried out in the home when one lives alone. Men and women adopt different stances and strategies (women as gatherers and men as hunters). For men, living alone is harder when it comes to everyday tasks in the home.	*E 13: A huge number of widowers have never even touched a dish in their lives (…) a man is not trained to do domestic tasks like a woman.* *E 14: If the woman is missing, it all goes to pot, but if the man is missing, the home is like this (…) she instinctively cares for the home. I do it because I have to.*
**The relationship between perceived perceptions of independence and over-estimating abilities**	Being independent and—for them, what is synonymous with this—being able to live alone are seen by more traditional Older people as not needing assistance with hygiene or meals. They tend to show off their abilities and to over-estimate them, while also hiding their losses. They consider them to be inherent in a person and associated with survival and keeping up the fight. If they can get by, they prefer not to ask for help or to hire help. They tend to accept situations and are dilatory when it comes to making changes. For other older people, their abilities are associated with intellectual skills, built up as a result of their training, work, life experiences and discipline. Adapting to change is part of being successful at aging. If they have aid or assistance at their disposal, they use it.	*E 13: I’ve always been like this, very independent and self-reliant. I don’t like to rely on anyone. I never carry even a parcel, I can’t carry any weight, my bag’s too much for me (…) everyone gets old, but you have to get there in an intelligent way; that is, without demands.* *E 16: Moral strength helps you to tackle any situation, together with discipline (…) I have conserved some not over-strict religious principles (…). It’s a base, a platform, an aid; that is, something to grab on to.*
**Category “Changes and Everyday Aspects of Domestic Life”**		
**CODE**	**DEFINITION**	**VERBATIM**
**Strategies to stay in the home**	Older people, especially the oldest, have an attitude of living today in the face of the non-existence of a future. Attitude, which is necessary in order not to anticipate losses and avoid planning for the future. Older people, aware that to a greater or lesser extent tomorrow will bring disability and illness, tend to live in the present. Their perception of the future modifies the value of time, there is no rush, the rhythm in old age is slower than that of an adult. After this stage there is no other. In known spaces, risks are minimized and points that require special attention are identified; In the house in which they have lived, the older person has a sense of security and control, regardless of architectural barriers. Older people show a tendency to reduce the demands on home maintenance and activities of daily living, as a way of compensating for changes in old age. The adaptation of the home is decisive in the way in which the older person seems to resolve the difficulties in daily life to satisfy basic needs.	*E 13: Student furniture, the cheapest I could find, and I set up a student-type flat that’s easy to clean (…). I looked for all the comforts, a lift, parquet, hot water and no dangers.* *E 3: Now I have a gadget for opening cans, like tins of tomatoes (…) often I don’t look at the expiry date (…) that box for the meter was put in, and my son-in-law took charge of doing it, and now they’ve brought me some papers that I had to sign that I told my granddaughter about…*
**Self-care as a way of continuing to live on one’s own**	Caring for oneself in necessary matters related to eating properly, taking prescribed medicine and doing physical activity. More traditional older people prefer to eat what they cook. They identify the beneficial properties of different produce and account for their reason for eating this type of food. They prefer not to have to cook. Physical activity/healthy leisure activities for more traditional older people mean taking a walk. More up-to-date older people do different types of physical activity (swimming, gym, cycling) as well as travelling. They also attend musical activities. They are interested in languages, discussion activities, or classes.	*E 16: I try and eat a good diet, with lots of vegetables (…) I try to be fairly prudent, it’s a bit like being an epicurean (…). Recipes, yes. Well, it’s here, written down on the page or if not, if it’s more complicated, I type it on the computer.* *E 2: I go to activities for the third age and to gym and memory training (…) that way, you’ve got a reason to say I’VE GOT to get up.*
**Category “Reliance on Social and Family Assistance”**		
**CODE**	**DEFINITION**	**VERBATIM**
**Traditional family: care is a moral duty**	More traditional older people who live alone consider the family to be by far the main support. Physical proximity is important. If this support is absent or not available, certain neighbors rank next in the support network. Older people with a traditional notion of the family believe that relatives have a moral obligation to care for them. They do not contemplate the possibility of hiring external carers or help in domestic tasks.	*E 2: Having family, so that you can live alone, is better, but not essential.* *E 8: Because nowadays children or daughters-in-law all work, you see, and they can’t look after you (…) because my daughters-in-law, one works until 7 p.m. since she’s seen the lie of the land and she’ll not get involved, and the other has what you might call a servant, so no way will she get involved helping out here! (...). If I go to the café—I’ve got friends there—if anything happens, they know they’ve got to help me.*
**Alternatives to traditional care**	Those with a more up-to-date approach to the family take a broader view to their support network, including friends, neighbors, unmarried partners and others. They believe that they can count on the support of a social and family network and hired services to meet their care needs. These older people do not believe that care is a family obligation.	*E 13: The neighbors keep an eye out. If they see the curtains are closed, they might come up and see if I’m ill (…) if my friends from the bars don’t see me—I spend half my life down there -, they ask about me.* *E 17: I’m very lucky. A lot of mothers or fathers haven’t much luck with their children (...). But my daughter says “You’ll NEVER, never go to another house. When you can’t think for yourself, you’ll come with me”.*
**Category “The Use of Social Services and Resources”**		
**CODE**	**DEFINITION**	**VERBATIM**
**Perceptions of healthcare professionals**	They regard attention by healthcare professionals as occasional and basically see them as the suppliers of medicines. They cannot identify the managing body that provides the relevant care and do not make a request for assistance. Relatives tend to act as a middleman in relations with healthcare professionals so that the older person does not have to travel to the health center so much.	*E 13: He writes the prescriptions but he never asks about me (…) No one’s come here, no one’s lent me a hand (…) I had to go twice to get the medicine.* *E 11: They just sign the prescriptions and, from time to time, the knee sometimes. I take my blood pressure and the nurse too, we usually coincide (…) but no, they haven’t taken records of my medical history or anything remotely like that.*
**Technology and tools to provide support for people**	Older people often act in a dependent way to show that they are dependent. Although they are aware of support systems that could improve their everyday lives, they do not feel capable of carrying out the complex formalities needed to request this assistance. When relatives are younger and available, they tend to take charge of most of the formalities needed to apply for help. The older people are unaware of the existence or availability of some support tools or systems and they are misled in knowing how suitable or not they would be. Call alert telecare is the best-known, most widely used tool, but the older people have different attitudes to it. Some consider it to be a convenient, easy-to-use device that guarantees greater security and others believe that it is not so convenient and that it mainly sets the family’s mind at ease.	*[Call alert telecare] I haven’t asked for anything because I don’t want to pay. What they offer isn’t worth it. I’ll use my mobile to ring if I’m ill or feeling bad.* *E 1: I asked for...I don’t know…whatever they would give me in May two years ago (…) “he can cope, he’s okay, he’s okay” (...). [Home help] The same home help that comes here goes to nine houses and finishes at 3 a.m.*
**Alternatives to their own home**	When the older person cannot go on living in their own home alone, they consider two possible alternatives: to live in an old people’s home, overcoming any negative connotations that these homes may have for people in general and for them, or going to live with a relative, generally their children. In this last case, whether there is enough room there or not can determine whether they have to take turns at living in two different carers’ houses. Whether the family has enough time or not to accompany the older person can determine the need for outside help.	*E 13: An attempt at living at a son’s house that ended in disaster, that simply wasn’t possible (…) of course, you have to remember that the home isn’t yours and so it’s very difficult.* *E 9: Sometimes you’ve got to go to an old people’s home because there are things you need (…) for my age, my illness, my sugar level (…) but while it’s still not urgent or necessary, I’m not going!*
